# RNAi-Based Functional Genomics in Hemiptera

**DOI:** 10.3390/insects11090557

**Published:** 2020-08-20

**Authors:** Ritesh G. Jain, Karl E. Robinson, Stephen J. Fletcher, Neena Mitter

**Affiliations:** Queensland Alliance for Agriculture and Food Innovation, Centre for Horticultural Sciences, The University of Queensland, Brisbane 4072, Queensland, Australia; r.jain1@uq.edu.au (R.G.J.); s.fletcher@uq.edu.au (S.J.F.); n.mitter@uq.edu.au (N.M.)

**Keywords:** RNAi, hemipteran insects, functional genomic studies, Hemiptera

## Abstract

**Simple Summary:**

RNA interference (RNAi) is a powerful strategy to understand the function of novel and critical insect genes. In this review, we highlight the pros and cons of using RNAi as a functional genomics tool, the range of applications and explore RNAi delivery approaches such as topical and carrier/nano-particle-mediated RNAi for silencing insect genes in Hemiptera. We explore factors contributing to observed variations in RNAi efficiency and possible solutions to improve RNAi based investigations. We briefly summarise and provide experimental insight on the key RNAi studies in agricultural hemipteran pests that will be applicable to lesser-studied hemipteran’s, as well as informing functional genomics studies across diverse insect pests.

**Abstract:**

RNA interference (RNAi) is a powerful approach for sequence-specific gene silencing, displaying tremendous potential for functional genomics studies in hemipteran insects. Exploiting RNAi allows the biological roles of critical genes to be defined and aids the development of RNAi-based biopesticides. In this review, we provide context to the rapidly expanding field of RNAi-based functional genomics studies in hemipteran insects. We highlight the most widely used RNAi delivery strategies, including microinjection, oral ingestion and topical application. Additionally, we discuss the key variables affecting RNAi efficacy in hemipteran insects, including insect life-stage, gene selection, the presence of nucleases, and the role of core RNAi machinery. In conclusion, we summarise the application of RNAi in functional genomics studies in Hemiptera, focusing on genes involved in reproduction, behaviour, metabolism, immunity and chemical resistance across 33 species belonging to 14 families.

## 1. Introduction

The Hemiptera (aphids, psyllids, whiteflies, leafhoppers, planthoppers, cicadas, moss bugs, and stink bugs) are the fifth largest insect order comprising more than 90,000 species, with highly diverse life histories and feeding habits. Functional genomics approaches have been increasingly used to investigate the molecular basis of important traits in many members of this order [1,2]. Conventional functional genomics studies in insects have been primarily based on forward genetics, with the function of interest first selected and the genes contributing to it subsequently identified [3]. The rapid progress of next-generation DNA sequencing has allowed researchers to assemble the genomes of several economically important hemipteran pests such as aphids [4] and whitefly [5]. With genomes and transcriptomes of many species becoming increasingly available, research focus has shifted to the identification of roles for genes of unknown function.

The application of reverse genetics, in which a gene is selected prior to discovering its function, represents a powerful approach when the DNA sequence of the insect is known [3]. Conventional reverse genetic approaches comprise random mutagenesis (chemical and transposable element mutagenesis) or targeted mutagenesis of the gene of interest (homologous gene recombination to generate null and hypomorphic mutations) to generate mutants with an observable phenotype. Although these methods are powerful in understanding the novel functions of genes, large-scale genetic screening via these approaches is challenging, time-consuming, and expensive [6]. Simpler, faster and inexpensive approaches for unveiling gene function in a selective and sequence-specific manner have evolved to utilise RNA interference (RNAi).

**Figure 1:**
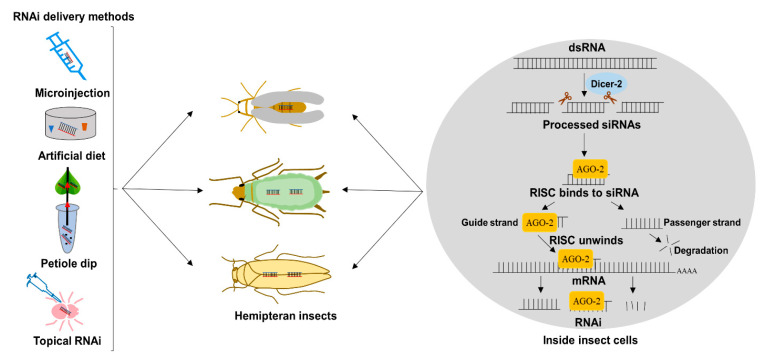
Schematic of RNA interference (RNAi) delivery strategies and RNAi mechanism in insects. In functional genomic studies in Hemiptera, dsRNA can be delivered by injection, diet feeding, petiole dipping and by topical application onto the target insect. Once the dsRNA is entered into the insect cell, dicer-2 recognises and cleave the dsRNA into siRNAs (21–24 nt). The siRNAs are then loaded in the RNA-induced silencing complex (RISC) that guides sequence-dependant degradation or translational inhibition of homologous mRNAs, resulting in RNAi-mediated silencing of the target gene.

RNAi is a highly conserved mechanism triggered by the introduction of sequence-specific double-stranded RNA (dsRNA) molecules, leading to target-specific endogenous gene silencing [7]. For an in-depth explanation of the RNAi mechanism (Figure 1), readers are directed to other reviews [8,9,10,11]. Since its discovery, a number of RNAi-based genomic studies exploring the function of individual genes and gene families involved in complex biosynthetic pathways of insects has exploded. Moreover, RNAi has been exploited to discover roles of homologous genes expressed in diverse species covering a broad spectrum of insect orders, providing critical insight into the evolutionary and developmental processes that have modelled these gene functions in insects [3].

**Figure 2:**
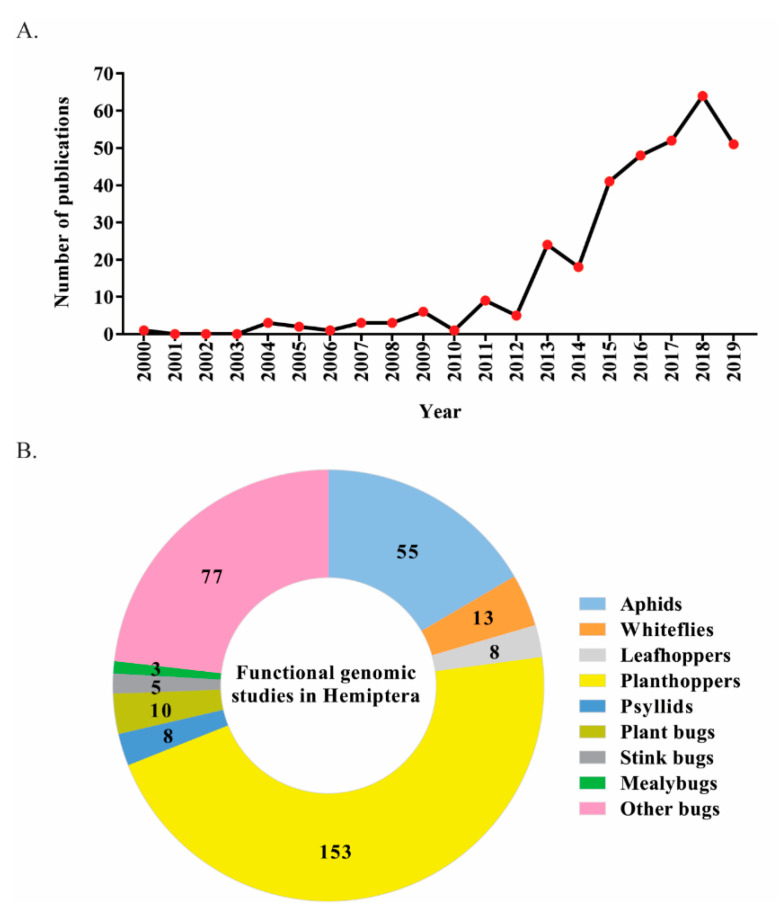
Dynamics of publications focused on RNAi-based functional genomics studies in major hemipteran pests. (**A**) The number of research publications for the past 20 years; (**B**) the number of functional RNAi studies in different hemipteran pests.

A critical survey of the literature demonstrates that induced RNAi has been successfully exploited for studying physiologically relevant hemipteran insect genes involved in embryogenesis, regeneration, development, reproduction, behaviour, virus-transmission, and insect resistance. Using the PubMed search engine (https://www.ncbi.nlm.nih.gov/pubmed/) with the queries “Hemiptera gene silencing”, “Hemiptera RNA interference”, “Hemiptera RNAi“ and “(scientific name of insect) RNAi”, we identified more than 329 research publications focused on RNAi-based functional genomics studies in hemipteran insects (Figure 2). These manuscripts demonstrate an increase of RNAi-based hemipteran functional genomics studies over the previous 20 years, most likely due to advances in transcriptomic and genomic information.

In this review, we discuss RNAi delivery methods, as well as key factors and challenges associated with variation in RNAi phenotypes observed in hemipteran insects. Furthermore, the current state of RNAi-based studies in gene function in Hemiptera has been summarised and discussed.

## 2. RNAi Delivery Strategies for Functional Genomics Studies in Hemiptera

The majority of RNAi-based functional genomics studies have utilised microinjection [12] and artificial diet [13] approaches to deliver dsRNA to hemipteran insects (Figure 1). In a limited number of studies, RNAi phenotypes were also successfully induced by topical application [14] and ingestion based methods such as transgenic plants [15], and detached stem dip [16], which are limited to plant-eating hemipteran insects.

### 2.1. Delivery by Microinjection

Microinjection is a classical method of dsRNA delivery to target insect tissues to initiate an RNAi response. It is considered the preferred approach for fundamental research and functional genomic studies and offers unique benefits by delivering precise amounts of RNAi molecules into egg, nymph and adults with ease and high efficiency. Further, microinjection allows RNAi molecules to reach the target tissue of interest or into the hemolymph, thereby circumventing potential barriers such as the integument, saliva and gut nucleases or periotrophic/microvillar membranes, which can hinder alternative feeding or soaking methods [17]. Microinjection has been successfully exploited to deliver RNAi molecules in several gene function studies in hemipteran insects including the pea aphid *Acyrthosiphon pisum* [18], whitefly *Bemisia tabaci* [19], and the glassy-winged sharpshooter, *Homalodisca vitripennis* [20]. However, microinjection does have disadvantages; it is delicate, laborious, and time-consuming [21,22], requires optimisation to fit with the developmental stage of the target insects and is generally limited to larger hemipteran insects and their eggs. Smaller insects such as psyllids [23] sustain significant inoculation damage, which affects their physiology and complicates the interpretation of results. Although injection-based delivery of RNAi is restricted to academic research, the technique remains extensively used in in vivo hemipteran functional genomics studies.

### 2.2. Delivery by Artificial Diet

A practical alternative to microinjection is the use of the artificial diet bioassay (AD), which is an efficient method of introducing RNAi effectors into the target insect body via ingestion [24]. AD overcomes several of the challenges and limitations encountered with microinjection [23] and is a convenient way of large-scale screening of RNAi targets in hemipteran insects [25]. For instance, the AD feeding-based approach has been successfully used to study functions of a wingless gene (*Wg*) in rice planthopper, *Sogatella furcifera* nymphs [13], doublesex (*Btdsx*) and transformer 2 (*Bttra2*) in adult *B. tabaci* [26] and *Orco* gene in the grain aphid, *Sitobion avenae* [27]. Despite many successful studies [28,29,30], AD does have disadvantages that researches must take into consideration, including (i) difficulty in determining the exact amount of dsRNA consumed by the insect [31], (ii) premature degradation of dsRNA through fungal and bacterial contamination, confounding phenotypic outcomes [32], and (iii) the requirement for relatively high dsRNA concentrations due to the potential degradation of dsRNA in the insect saliva and gut by nucleases prior to transport of dsRNA to target tissues [21,33]. This last issue can be addressed by encapsulation of dsRNA with cationic liposome as similarly done in the neotropical seed-sucking stink bug *Euschistus heros* [34], and the fruit fly *Drosophila melanogaster* [35] to enhance RNAi effects. Liposome-encapsulated dsRNA has been demonstrated to be more stable in insect saliva compared to non-encapsulated dsRNA [34].

### 2.3. Delivery of dsRNA by Topical Application to Insect and Plants

The topical application of dsRNA to the insect body has been exploited for gene function studies in hemipteran insects [14]. The drawbacks of microinjection and AD-mediated ingestion of dsRNA facilitated the development of topical RNAi, which allow for the investigation of RNAi efficiency in a dose—and time-dependent manner. The technique also permits the study of functionally diverse and/or related genes in multiple hemipteran species simultaneously, thereby saving time and cost [36]. The topical application of one dsRNA construct targeting five closely related cytochrome P450 of Asian citrus psyllid, *Diaphoriana citri* resulted in reduced resistance to imidacloprid [37], demonstrating topical RNAi could be used to target multiple genes in hemipteran insects simultaneously. Similarly, a topically applied cathepsin-L cysteine dsRNA on sunn pest, *Eurygaster integricep*, penetrated the cuticle and affected nymphal stage development [14], supporting the functional role of *cys* in insect development and moulting. Although these studies indicate that topical RNAi is effective in some species, the method is challenging in some insects due to the avoidance of barriers such as the cuticle. Thus, formulation additives such as penetrant and/or nanoparticles could be used to improve the penetration of dsRNA through the cuticle.

Interestingly, the topical delivery of dsRNA using nanoparticle carriers enhances the stability and uptake of dsRNA via the cuticle. For instance, a droplet of a formulation comprising dsRNA specific to mammalian von Willebrand factor homologous hemocytin, detergent and nanocarrier were applied on the notum of the soybean aphid, *Aphis glycines*, with the dsRNA subsequently penetrating the cuticle and spreading throughout the body [38]. Similarly, siRNAs specific to the carotene dehydrogenase (*tor*) and branched-chain amino acid transaminase gene *bcat* were complexed with nanoparticles and delivered to three aphid species through aerosolisation using a nebuliser/compressor, which increased the efficacy of gene silencing and the associated phenotypes [39]. These studies suggest that the nanoparticle-mediated delivery of dsRNA could be a candidate to enhance RNAi efficiency in gene function studies in hemipteran insects.

The exogenous application of dsRNA molecules to plants to silence insect genes upon predation has shown promising outcomes. It is considered a viable and practical approach because it does not alter the plant’s genome and can be tailored in a short duration of time. In a study on hemipteran insects, authors have successfully delivered dsRNA molecules in two sap-feeding pests including psyllids and leafhoppers using non-transformative approaches such as foliar spray, trunk injection or root drenching [40]. In another study, the delivery of dsRNA specific to juvenile hormone acid methyltransferase (JHAMT) and vitellogenin (Vg) genes of the brown marmorated stink bug *Halyomorpha halys* via soaking vegetables was reported to reduce the target gene expression of insect significantly [41]. These studies indicate that the exogenous application of dsRNAs could be an attractive alternative for silencing target genes of insects in crop protection sense and a more natural system to research functional genomics in hemipteran insects.

## 3. Variables Affecting RNAi Phenotypes in Functional Genomics Studies in Hemipteran Insects

Although several studies have reported successful application of RNAi for defining gene function in hemipteran insects, RNAi efficiency among insect species varies significantly. These variations are reliant on several critical factors such as dsRNA delivery and processing, the presence of nucleases, and cellular uptake of dsRNA [33].

### 3.1. Life-Stage of Insect

The life-stage of the insect is often a key consideration in RNAi-based functional genomics studies. Although adult stage insects are often more easily handled than nymphal stages, gene silencing effects are generally more prominent in the early and immature development stages (eggs and nymphs) [22,42]. As an example, *D. melanogaster* larvae displayed susceptibility to RNAi after being injected with DIAP1 (Drosophila Inhibitor of Apoptosis Protein (1)) dsRNA however, adults were resistant [43]. The possible explanation for this variation in RNAi susceptibility could be due to the higher expression of the selected target gene or nuclease encoding genes in the adult than the larval stage of the insect, making them resistant to RNAi-mediated silencing. In contrast, it is not always possible to study the role of specific genes via injection of dsRNA into insect embryos, for instance, RNAi in southern green stinkbug *Nezara viridula* is only effective in nymphs as embryos are resistant to microinjection [44]. Thus, parental RNAi (pRNAi), a gene silencing response where the interference effect is observed in the offspring of the treated species [45], remains the only possible method to study these genes in *N. viridula*.

### 3.2. Target Gene Selection

The selection of target genes for functional genomics studies is of paramount importance, contributing to the efficiency of RNAi and resulting phenotypes in the target insect. Factors that need to be considered include; (i) Tissue-specific gene expression. In piercing-sucking insects, systemic spreading of RNAi is limited due to the absence or lack of function of RNA directed RNA polymerase (RdRP), which is required for the synthesis of secondary siRNAs and RNAi amplification [46]. This phenomenon suggests that in the case of AD, the midgut cells are the preferential target site where an RNAi response could be triggered [47]. Spatial and temporal knowledge of target gene expression is considered critical for both the selection of the target gene and the delivery method used. (ii) Protein stability and turnover rate. The time lag between the initiation of the RNAi response and gene silencing is greatly influenced by protein half-life and mRNA turnover [48]. For instance, the injection of dsRNA targeting *calreticulin* and *cathespsin-L* in *A. pisum* caused a decrease in target gene expression by 35–41% at one, two, three, and five days post-injection (dpi). However, dsRNA did not affect target gene expression seven dpi [49], which could be an indication of a rapid turnover of the corresponding proteins. Therefore, a suitable RNAi gene for functional genomics studies should be moderately expressed in the desired tissue, be able to generate an mRNA transcript with a rapid turnover rate and be translated into a protein with low stability [48]. (iii) The abundance of target mRNA. The level of mRNAs in the target tissue is an essential factor for successful RNAi phenotypes. Target genes expressed at lower levels may require less dsRNA for effective gene silencing. For instance, studies comparing gene expression and dsRNA dosage rates for induction of RNAi are limited; however, researchers have demonstrated *H. halys* nymphs exposed to 0.017 µg/uL dsRNA targeting JHAMT and vitellogenin (Vg) genes caused a 2.2-fold reduction in the abundance of Vg transcript, with JHAMT expression not affected when compared to controls [41]. In contrast, increasing the dsRNA concentration to 0.067 µg/uL, a 4.5-fold depletion of the amount of JHAMT mRNA was observed and suggests variations in knockdown efficiency among target genes is a function of the abundance of dsRNA applied and/or the relative expression of the target gene in the insect.

### 3.3. Nucleases

For successful RNAi phenotypes, the rapid transport and cellular uptake of intact dsRNA is a vital step before enzymatic degradation by dsRNases in the saliva, haemolymph, and midgut that can destroy dsRNA effectors [33]. For instance, injection of dsRNA targeting *IAP* (inhibitor of apoptosis) of the tarnished plant bug *Lygus lineolaris* caused significant gene silencing; however, oral delivery of IAP dsRNA did not affect target transcript level and was attributed to dsRNA degradation by salivary dsRNase [50]. In a similar study on the aphid *Myzus persicae*, researchers demonstrated that endonucleases in the midgut were responsible for insensitivity of the aphids to orally delivered dsRNA [51]. Thus, targeting nucleases for improving RNAi phenotypes in gene function studies in hemipteran insects would be an attractive idea. Interestingly, co-administration or stacking of dsRNA targeting two nucleases (dsRNases) encoding genes of *B. tabaci* depleted the target mRNA level and improved RNAi efficiency [52].

### 3.4. Core RNAi Machinery

Following internalisation of dsRNA into cells, the first step for triggering an RNAi-mediated phenotype is the recognition of dsRNA by the core RNAi machinery. Several factors relating to core RNAi machinery are responsible for phenotypic variation in hemipteran RNAi studies; (i) Copy number of core RNAi genes. More than one copy of core siRNA enzyme-encoding genes (*Dcr2, Ago2, R2D2*) suggests a higher RNAi efficiency in some species [53]. Conversely, single copies of core RNAi genes are often responsible for an inadequate RNAi response. As an illustration, the superior RNAi response in *Tribolium castaneum* as compared to *Drosophila melanogaster* is purported to be due to an additional one copy of *Ago2* and *R2D2* [33,54]. One or more copies of core RNAi genes such as *dicer2* and *Ago2* have been identified in some hemipteran species (Table 1) such as *Diuraphis noxia, A. pisum, Nilaparvata lugens, Diaphorina citri, Aphis glycines* and *B. tabaci*, [33] which all show comparatively robust RNAi phenotypes. However, the copy number of core RNAi genes alone does not explain the variation in RNAi phenotypes observed among hemipteran pests and remains a phenomenon to be investigated.

(ii) Expression of core RNAi genes. The variation in expression and function of RNAi associated genes in different tissues and species likely also contributes to the functionality of the RNAi machinery in insects [63]. For instance, RNAi efficiency in transgenic *D. melanogaster* with multi-copy overexpression of a *Dcr2* transgene was increased compared to wild-type control lacking extra *Dcr2* transgenes. These observations demonstrate that the overexpression of *Dcr2* enhances the RNAi efficiency in the target insects [64]. On attempting to silence the *Dcr2* gene of *B. germanica* via dsRNA injection, researchers unexpectedly observed increased *Dcr2* expression post-exposure to *Dcr2* dsRNA, that remained elevated for one day post-exposure [65]. This study further suggests that the Dicer-2 is a putative sensor of viral infections and expression of the core RNAi components influences the RNAi response. (iii) The redundancy of RNAi pathways. Endogenous gene expression in insects is regulated by three RNAi pathways: the siRNA pathway, the miRNA pathway and the piRNA pathway. Enzymes in these pathways responsible for triggering RNAi activity may contribute to variation in RNAi phenotypes among insects [33]. For example, the argonaute enzyme (AGO) from either of the three RNAi pathways can carry out the processing roles of dsRNAs in *Bombyx mori, Leptinotarsa decemlineata,* and *D. melanogaster* [33]. Taken together, the factors associated with core RNAi machinery must be carefully considered before exploiting RNAi for functional genomics studies in hemipteran insects. (iv) Systemic RNAi. In insects, the red flour beetle, *Tribolium castaneum*, *systemic RNA interference deficient-1* (*Sid1*) is responsible for translocating the RNAi signal throughout the body [54]. Although studies have demonstrated the presence of *Sid 1* homologs in several hemipteran insects, such as soybean aphid *Aphis glycines* [56], the involvement of this protein in translocation of the RNAi signal is still to be elucidated. The lack of *Sid1*, or its weak level of temporal expression in tissues, would indicate an absence of systemic RNAi and thereby lower RNAi efficiency in functional genomics studies.

## 4. Applications of RNAi in Functional Genomic Studies of Hemiptera Insects

In hemipteran insects, functional genomic studies using gene silencing have been reported in [14] families and [33] species, and representative gene functions from these insect species have been summarised in Table 2 and are discussed below.

### 4.1. Parental RNAi

Parental RNAi (pRNAi) is a useful method for examining early embryogenesis and is a feasible approach for studying RNAi in hemipteran insects whose eggs are not viable or sustain a significant injury after dsRNA injection [112]. pRNAi effects have been reported in several hemipteran species such as *Sitobion avenae* [113], *Rhodnius prolixus* [92], *Myzus persicae* [15]. In *R. prolixus*, dsRNA specific to the salivary hemoprotein nitrophorin was injected into fifth instar nymphs, and subsequently, the RNAi effects were monitored at various developmental stages. Nitrophorin mRNA reduction significantly persisted in all life stages of the insect for more than seven months of post-dsRNA injection [92]. This study supports the persistence of the RNAi response over multiple generations, which could be essential for RNAi-mediated control of hemipteran insects. In the aphid *M. persicae,* the silencing of effector genes *MpC002* and *Mp2,* which are purported to be essential in aphid-plant interactions, resulted in significant knockdown in nymphs born from females ingesting dsRNA expressed in transgenic *Arabidopsis thaliana* plants [15]. The RNAi activity not only persisted longer in offspring than parents, but the impact of RNAi persisted over three aphid generations. In *N. cincticeps*, the injection of dsRNA targeting *lacase-2* in female insects resulted in significant gene silencing in nymphal offspring [67]. Although these studies suggest that parental RNAi can be utilised to investigate the gene functions and germline effects in the offspring of parental females, further research is warranted to investigate the stage of female reproductive development where pRNAi could be most effective.

### 4.2. Embryonic RNAi

In hemipteran insects, embryonic RNAi (eRNAi) is preferably employed to study genes responsible for oogenesis, with embryo injection extensively used to investigate the functions of genes involved in embryo development and cellular differentiation [50]. For example, previous studies in *Drosophila* have shown that the homeotic complex (*hox*) genes are required for segmental identity during embryogenesis [114]. In *Oncopeltus fasciatus*, *hox* genes consist of *Ultrabithorax*, *Abdominal-A*, *Abdominal-B,* and *Antennapedia* [115]. Here, the silencing of *Antennapedia* caused a transformation of the thoracic appendages toward antennal morphology, suggesting that *Antennapedia* is necessary for the differentiation of the thoracic segments. RNAi-mediated knockdown of *Ultrabithorax* resulted in the formation of an ectopic pair of leg-like appendages, indicating that *Ultrabithorax* is a suppressor of leg development. Likewise, RNAi of *Abdominal-A* and *Abdominal-B* caused defects in ectopic appendages and a loss of characteristics in posterior abdominal segments, respectively. Similarly, in *O. fasciatus,* the function of the gap gene *hunchback* in embryogenesis was investigated using RNAi [116]. Silencing of *hunchback* using dsRNA microinjection in embryos resulted in impaired development of the gnathic and thoracic regions and increased the posterior compaction of the embryos [116]. These observations confirmed that *hunchback* is essential to suppress abdominal identity in these regions and is vital for proper germband growth and segmentation in *O. fasciatus*. Additionally, if a target gene is essential for egg formation then silencing of these genes may prevent the generation of eggs, in such cases, eRNAi could be a preferred approach over pRNAi to obtain RNAi phenotypes in embryos. Thus, eRNAi would be indispensable to determine gene function during embryogenesis.

### 4.3. Post-Embryonic RNAi

The most widely studied gene functions during post-embryonic development in hemipteran insects are related to metamorphosis, ecdysis and moulting [117]. For instance, the oral delivery of dsRNA targeting the ecdysone receptor (*EcR*) and ultraspiracle protein (*USP*) significantly reduced the survival and fecundity of *S. avenae* [77], confirming that *EcR* and *USP* genes play an essential role in growth and development. Further, injection of cathepsin-L dsRNA to *A. pisum* silenced the target mRNA level in the body carcass, which induced alterations in the body morphology of the aphid [118]. Here, the feeding of cathepsin-L dsRNA also triggered target gene knockdown in the gut and resulted in morphological abnormalities, suggesting a function of *cathepsin-L* in aphid moulting. In *A. pisum*, injection of chitin synthase (CHS) dsRNA into fourth-instar nymphs resulted in impaired development and deformity among newly born nymphs [119], revealing the role of *CHS* gene in the nymphal growth and embryonic development. Taken together the findings of these post-embryonic RNAi studies could provide valuable insight on the molecular basis of morphological diversity in various hemipteran pests.

### 4.4. Life Stage-Specific RNAi

RNAi has been extensively utilised in functional genomics due to its flexibility to specifically target developmental stages of the insect [120]. RNAi studies in *S. furcifera* illustrated the potential role of ryanodine receptors (RyRs) in insecticide uptake and toxicity in second instar nymphs [121]. Here, following dietary ingestion of *RyRs* dsRNA, exposure of nymphs to chlorantraniliprole insecticide led to a decrease in mortality. This study illustrated that RNAi could be an alternative to classical in vivo and in vitro metabolism studies identifying resistance mechanisms in immature life-stages of insects [122]. RNAi has also been exploited to study the role of genes critical for the nymph-adult transition. For instance, suppression of insulin receptor genes (*AcInR1* and *AcInR2*) in the fourth instar *Aphis citricidus* via feeding resulted in the transition of nymphs to abnormal adults with deformed wings, confirming that *AcInR1* and *AcInR2* are necessary for aphid wing development [123]. Though the authors did not elucidate the functional mechanisms of these genes in wing development, the study provided valuable clues relevant to nymph-adult transition in aphid that could be further explored in future studies.

### 4.5. RNAi in Studying Reproduction-Related Genes

RNAi-based studies for exploring the functions of sex determination cascade related genes can provide insight into the development of the male and female lineages in hemipteran insects. In *B. tabaci*, knockdown of the *dsx* gene caused deformation of the male anal stylet and reduction in the expression of vitellogenin in females [26]. These observations illustrate that *dsx* is involved in sex determination and male genitalia formation in *B. tabaci*, which can be exploited for studies of sex determination in other hemipteran and haplodiploid species. Vitellogenin (Vg) is the most crucial protein in vitellogenesis, which is produced in the insect fat body, then transported to the hemolymph and integrated into the oocytes [3]. RNAi has allowed the silencing of Vg expression in bedbug *Cimex lectularius* females, resulting in a significant reduction in egg production and atrophied ovaries [124]. This study demonstrated the potential role of Vg in reproduction and survival and provided a promising gene target for RNAi-based management of the bedbug. The outcomes of these studies can be useful for future research aiming to understand how hemipteran insects produce plentiful offspring and how their reproductive capabilities can be effectively suppressed or disrupted using RNAi-based strategies.

### 4.6. RNAi in Behavioural Biology

The study of circadian rhythm, an endogenous timing system, is crucial to gain knowledge about the insect’s daily activities and behaviours, such as feeding, eclosion, mating and oviposition. Although the circadian clock has been extensively studied in hemipteran insects, few RNAi-based studies have been conducted to unveil the roles of core circadian genes (*period*, *timeless*, and *cycle*). For example, knockdown of the *timeless* (*tim*) via dsRNA injection in *Laodelphax striatellus* impaired the adult’s locomotor activity, behaviour in continuous darkness, and the timing of normal adult emergence [88]. These results provided knowledge regarding the endogenous circadian network responsible for the behavioural and physiological rhythms of this insect. RNAi of *Clock* abolished the circadian rhythm of cuticle layer deposition and blocked ovarian development regardless of day-length conditions in the bean bug *Riptortus pedestris* [93]. These studies explored the potential role of circadian genes, providing critical information to understand the mechanism involved in the adaptation of insects. Further behavioural studies encompassing including how the insect senses its surrounding, pathways to processing the signal, and systems that allow the insect to act on the sensory information could be critical for effectively managing hemipteran pests.

### 4.7. RNAi in Exploring Biosynthetic Pathways

In hemipteran insects, the most common pathways studied using RNAi are those involving chitin, eye pigment, tanning and sex pheromones, as well as critical processes involved in insect development and survival. As an illustration, RNAi-mediated silencing of the chitin synthase gene (*CHS*) in *A. pisum* via injection or plant feeding caused moulting deformities in exposed and newborn nymphs [119]. Further highlighting that *CHS* is a vital component of the insects’ exoskeleton and is crucial for aphid growth and embryonic development. Moreover, RNAi-based studies have been conducted to determine the relationship between olfactory proteins and the behaviour of insects in response to detection of the chemicals and compounds. For example, silencing of odorant-binding proteins (OBPs) in *Adelphocoris lineolatus* reduced antennal responses to sex pheromones and plant volatiles, suggesting that OBPs plays a role in food and mate identification [98]. These promising outcomes may serve as a foundation for future investigations that aim to target insects’ olfactory system to interfere with the mate-seeking and host-locating behaviour of insects.

### 4.8. RNAi in Immunity-Related Genes

Hemipteran insects have a specialised defence system based on cellular and humoral reactions for fighting pathogens, and RNAi has helped uncover several components of these mechanisms. To give an example, downregulation of *hexamerin* in leafhopper *Circulifer haematoceps* via RNAi decreased phenoloxidase activity increasing insect mortality by when challenged by the bacterium *Spiroplasma citri* [104]. The results suggest that hexamerin is vital for protecting the leafhopper against bacterial infections. In general, insect immunity is controlled by the immune deficiency pathway (IMD) and Toll signalling pathway that targets bacteria and fungi. RNAi against selected components of these pathways in hemipteran insects helped elucidate the defence mechanism. Similarly, in *Sogatella furcifera*, silencing of *Akirin* via injection depleted expression of the nuclear transcription factor (NF-kB) after bacterial challenge [125], suggested *Akirin* regulates the immune response of insects via IMD and Toll pathways. Overall, these studies demonstrated the potential of RNAi to understanding hemipteran pest-pathogen interaction and host responses which could focus efforts to control vector-borne phytopathogens by reducing transmission capability of these insects.

### 4.9. RNAi Studies to Uncover the Mechanism of Resistance Against Chemicals

In hemipteran RNAi, detoxification genes are a focus of studies due to their involvement in insecticide resistance. Overexpression of detoxification genes can lead to higher enzymatic activity and the occurrence of resistance [126]. The major detoxification enzymes in the insect midgut are cytochrome P450, carboxylesterases and glutathione S-transferases (GST) [110]. Feeding of CYP dsRNAs to *D. citri* reduced the expression of target mRNA and enhanced the toxicity of imidacloprid against psyllids [108], indicating that CYP genes are involved in the metabolism of this insecticide. Injection of dsRNA targeting acetylcholinesterase 1 (*Ace1*) and acetylcholinesterase 2 (*Ace2*) into *Rhopalosiphum padi* and *Sitobion avenae* resulted in significant downregulation of the target gene and increased the toxicity of aphids to pirimicarb and malathion [111]. This study demonstrates that Ace1 is preferentially a cholinergic enzyme and is the primary target site of these insecticides, whereas Ace2 is less critical for toxicological functions as compared to Ace1.

## 5. Conclusions and Future Perspectives

Over the past two decades, RNAi has emerged as a valuable tool for functional genomic studies targeting critical genes of hemipteran pests. Previous RNAi studies in Hemiptera have reported several factors that contribute to the variations in RNAi efficacy between different species. While many studies are successful, these factors may lead to only partial or no gene silencing failing to induce RNAi phenotypes in some cases. Therefore, further studies are needed to identify additional genes, mechanisms and biological pathways responsible for the observed variations in RNAi efficacy. Comparative RNAi-based functional genomics studies focusing on core RNAi genes in different hemipteran species could also provide valuable insight into the mechanism responsible for RNAi variations in hemipteran insects.

In recent years, RNAi delivery strategies such as foliar spray and carrier particles have been developed for gene silencing in pests and pathogens and are approaches that could be an applicable alternative for delivering RNAi molecules in functional genomics studies. These promising approaches may reduce the cost, time, and increase reliability associated with the current delivery methods, and may open new possibilities to investigate functions of genes of interest.

The discovery of RNAi has allowed researchers to perform functional genomic screening in several orders of insects. However, several technologies have become available to undertake functional genomic investigations and before selecting RNAi, one must consider the pros and cons of RNAi over recently developed technologies, such as CRISPR. CRISPR-Cas9 mediated gene editing is a highly efficient tool for gene modification however, modifies/mutates at the genomic level where RNAi modifies at the transcriptional level leaving the gene intact. Additionally, CRISPR-Cas9 knockout/modification of essential insect genes will be lethal in most instances providing only limited information regarding gene function other than “critical to survival” of the insect. In such a scenario, RNAi-mediated knockdown of gene transcripts can offer greater insight into the target gene function/effect on phenotype. Secondly, the transient nature of RNAi knockdowns can be used to validate phenotypic effects since the natural restoration of the target protein level in the organism restores function. Both CRISPR-Cas9 and RNAi approaches to functional genomic investigations suffer from off-target effects that can mutate/silence unintended genes/transcripts. Therefore, bioinformatics-based design of dsRNA molecules to minimise potential off-target effects should be a rigorous undertaking prior to the use of either technology, however, in our opinion, RNAi is the simplest method of eliciting a phenotypic effect, both logistically and functionally, and is the preferred tool for functional genomics studies in hemipteran insects.

The application of RNAi to study the gene function and to explore interactions between genes has provided invaluable insights into evolutionary and developmental processes in hemipteran insects. RNAi-based studies have also allowed researchers to unveil novel functions and roles of several hemipteran insect genes involved in growth, development, feeding, behaviour, virus-transmission, and insecticide resistance. The outcome of these studies can further facilitate comparative RNAi-based studies in other lesser studied hemipteran insects, leading to further critical insight into their evolution of vital processes such as host-insect interaction, feeding behaviour, and reproduction.

## Figures and Tables

**Table 1:** Overview of reported core RNAi genes in hemipteran insects.

Insect Species	No. of Core RNAi Genes			Reference
	*Dcr2*	*R2D2*	*Ago2*	
*Acyrthosiphon pisum*	1	1	1	[55]
*Aphis glycines*	1	1	1	[56]
*Bemisia tabaci*	1	1	1	[57]
*Diuraphis noxia*	1	1	2	[58]
*Diaphorina citri*	1	0	1	[59]
*Halyomorpha halys*	1	0	1	[60]
*Myzus persicae*	1	1	2	[51]
*Nezara viridula*	1	2	2	[61]
*Nilaparvata lugens*	1	1	1	[62]

**Table 2:** Application of RNAi in functional gene studies in hemipteran insects.

RNAi Studies	Family	Insect Species	Target Gene and Studied Functions	Ref
Parental RNAi	Aphididae	*Sitobion avenae*	Zinc finger protein *SaZFP*	[66]
		*Myzus persicae*	Salivary proteins *MpC002, MpPInto2*	[15]
	Cicadellidae	*Nephotettix cincticeps*	Role of *Laccase-2* in the cuticle pigmentation	[67]
	Pentatomidae	*Euschistus heros*	Chromatin-remodeling ATPases, *brahma, mi-2, iswi*, parental gene silencing	[68]
	Reduviidae	*Rhodnius prolixus*	*piwi* orthologs, role in oogenesis	[69]
Embryonic RNAi	Delphacidae	*Nilaparvata lugens*	*Hox3-like* gene involved in embryonic development	[12]
	Aphididae	*Acyrthosiphon pisum*	Heat shock protein 83 (HSP83) crucial in fecundity and embryogenesis	[70]
			Phenylalanine hydroxylase important for embryonic development	[71]
	Lygaeidae	*Oncopeltus fasciatus*	DNA methyltransferase 1 *(Dnmt1)* essential for egg production and embryo viability	[72]
			*Zen* gene, role in mid embryogenesis	[73]
	Reduviidae	*Rhodnius prolixus*	Heme-binding protein, role in embryogenesis	[74]
Post-embryonic RNAi	Lygaeidae	*Nysius plebeius*	*Ultrabithorax* require for bacteriocyte development	[75]
		*Oncopeltus fasciatus*	*Sex combs reduced (Scr),* role in wing suppression and wing program	[76]
	Aphididae	*Sitobion avenae*	Ecdysone receptor (*EcR*) and ultraspiracle (*USP*) essential in growth and development	[77]
	Scutelleridae	*Eurygaster integriceps*	*Cysteine* gene involved in growth and development	[14]
	Pentatomidae	*Halyomorpha halys*	*Sex combs reduced (Scr)* involve in development	[78]
Regeneration-dependant RNAi	Delphacidae	*Nilaparvata lugens*	*Decapentaplegic* gene, role in wing vein development and wing morph transformation	[79]
			Distal-less gene homologue, *NlDll,* role in leg development and wing structure	[80]
		*Sogatella furcifera*	Role of *Wingless* gene (*Wg*) in the development and growth of wings	[13]
		*Laodelphax striatellus*	Ecdysone receptor involved in wing morphogenesis and melanisation	[81]
	Liviidae	*Diaphorini citri*	Abnormal wing disc (*awd*) gene involved in wing development and metamorphosis	[82]
RNAi in studying reproduction-related genes	Delphacidae	*Nilaparvata lugens*	S-Adenosyl-l-methionine-dependent methyltransferases (SAMMTases), regulates reproduction	[83]
		*Sogatella furcifera*	Carboxylesterase precursor (*EST-1*), role in fungicide suppressed reproduction	[84]
	Reduviidae	*Triatoma infestans*	*Vitellogenin* Gene, role in ovipostion	[85]
	Aphididae	*Aphis citricidus*	Vitellogenin (*Vg*) and Vg receptor, Role in development and reproduction	[16]
	Aleyrodidae	*Bemisia tabaci*	Role of *doublesex* gene in sex determination	[26]
	Cimicidae	*Cimex lectularius*	Chromatin remodelling gene *Brahma* function in reproduction and survival	[86]
	Pyrrhocoridae	*Pyrrhocoris apterus*	Methoprene-tolerant (*Met*) involve in reproduction and development	[87]
RNAi in behavioural biology	Delphacidae	*Laodelphax striatellus*	Odorant-binding proteins, role in host-seeking behaviour	[33]
			*Timeless* gene crucial for circadian rhythms	[88]
	Cicadellidae	*Nephotettix cincticeps*	*Troponin C* involve in behaviour and fitness	[89]
		*Empoasca vitis Göthe*	*Opsin* genes are critical for host orientation behaviour	[90]
	Aphididae	*Acyrthosiphon pisum*	*Neuropeptide F* regulates feeding behaviour	[91]
		*Sitobion avenae*	*SaveOrco* gene, role in aphid’s response to pheromones	[27]
	Reduviidae	*Rhodnius prolixus*	*Nitrophorins,* reduced anticoagulant activity and poor feeding behaviours	[92]
	Alydidae	*Riptortus pedestris*	Role of *Clock* gene in the circadian rhythm	[93]
RNAi in exploring biosynthetic pathways	Reduviidae	*Platymeris biguttatus*	Tyrosine hydroxylase (*TH*), *yellow*, arylalkylamine-N-acetyltransferase (*aaNAT*), role in pigmentation	[94]
	Aphididae	*Aphis gossypii*	Farnesyl diphosphate synthase (*FPPS*), role in the biosynthesis of alarm pheromone	[95]
	Rhopalidae	*Jadera haematoloma*	Forkhead-box O (*FoxO*) critical for the evolution of polyphenism	[96]
	Delphacidae	*Laodelphax striatellus*	Forkhead-box O (*FoxO*) control hormone-mediated signalling pathway in nymphal diapause	[97]
	Miridae	*Adelphocoris lineolatus*	Odorant binding proteins play a role in the identification of volatile compounds and sex pheromones	[98]
		*Lygus lineolaris*	*Polygalacturonase* gene mainly express in salivary gland demonstrating that PGs are salivary enzyme	[99]
		*Lygus hesperus Knight*	*Cardinal* gene is essential for eye colouration	[100]
		*Adelphocoris suturalis*	*Desaturase-like genes*, role in sex pheromones biosynthetic pathway	[101]
RNAi in immunity-related genes	Pentatomidae	*Plautia stali*	Immune deficiency (IMD) pathway genes, role in controlling arrays of microbes	[102]
	Aleyrodidae	*Bemisia tabaci*	A defensin-like antimicrobial peptide is involved in begomovirus infection	[103]
	Reduviidae	*Rhodnius prolixus*	Immune-deficiency pathway (IMD) regulates the activity of the antimicrobial peptide	
	Cicadellidae	*Circulifer haematoceps*	*Hexamerin* gene is required for immune response and survival against the bacterium	[104]
		*Graminella nigrifrons*	Peptidoglycan recognition proteins involved in immune response and pathogen transmission	[105]
		*Recilia dorsalis*	Caspase is involved in promoting virus infection	[106]
RNAi studies to uncover the mechanism of resistance against chemicals	Delphacidae	*Nilaparvata lugens*	Cytochrome P450 (*CYP*) genes involve in nitenpyram resistance	[107]
	Liviidae	*Diaphorini citri*	*CYP* knockdown increased susceptibility to imidacloprid	[108]
	Aphididae	*Aphis gossypii*	*UDP-glycosyltransferases (UGTs),* role in imidacloprid resistance	[109]
			*Carboxylesterase* silencing led to increased sensitivity to organophosphorus	[110]
		*Rhopalosiphum padi*	*Acetylcholinesterase* gene is responsible for resistance to pirimicarb and malathion	[111]

## References

[1] Panfilio K.A., Angelini D.R. (2018). By land, air, and sea: Hemipteran diversity through the genomic lens. Curr. Opin. Insect Sci..

[2] Panfilio K.A., Jentzsch I.M.V., Benoit J.B., Erezyilmaz D., Suzuki Y., Colella S., Robertson H.M., Poelchau M.F., Waterhouse R.M., Ioannidis P. (2019). Molecular evolutionary trends and feeding ecology diversification in the Hemiptera, anchored by the milkweed bug genome. Genome Boil..

[3] Bellés X. (2010). Beyond Drosophila: RNAi In Vivo and Functional Genomics in Insects. Annu. Rev. Èntomol..

[4] Legeai F., Shigenobu S., Gauthier J.-P., Colbourne J.K., Rispe C., Collin O., Richards S., Wilson A.C.C., Murphy T., Tagu D. (2010). AphidBase: A centralized bioinformatic resource for annotation of the pea aphid genome. Insect Mol. Boil..

[5] Chen W., Hasegawa D.K., Kaur N., Kliot A., Pinheiro P.V., Luan J., Stensmyr M.C., Zheng Y., Liu W., Sun H. (2016). The draft genome of whitefly *Bemisia tabaci* MEAM1, a global crop pest, provides novel insights into virus transmission, host adaptation, and insecticide resistance. BMC Boil..

[6] Lin S.-C., Chang Y.-Y., Chan C.-C. (2014). Strategies for gene disruption in Drosophila. Cell Biosci..

[7] Vogel E., Santos D., Mingels L., Verdonckt T.-W., Broeck J.V. (2019). RNA Interference in Insects: Protecting Beneficials and Controlling Pests. Front. Physiol..

[8] Lim Z.X., Robinson K.E., Jain R., Chandra G.S., Asokan R., Asgari S., Mitter N. (2016). Diet-delivered RNAi in Helicoverpa armigera—Progresses and challenges. J. Insect Physiol..

[9] Bally J., Fishilevich E., Bowling A.J., Pence H.E., Narva K.E., Waterhouse P.M. (2018). Improved insect-proofing: Expressing double-stranded RNA in chloroplasts. Pest Manag. Sci..

[10] Yu X.-D., Liu Z., Huang S.-L., Chen Z.-Q., Sun Y., Duan P.-F., Ma Y.-Z., Xia L.-Q. (2016). RNAi-mediated plant protection against aphids. Pest Manag. Sci..

[11] Whitten M.M.A., Dyson P.J. (2017). Gene silencing in non-model insects: Overcoming hurdles using symbiotic bacteria for trauma-free sustainable delivery of RNA interference. Bioessays.

[12] Ren Z.-W., Zhuo J.-C., Zhang C.-X., Wang D. (2018). Characterization of NlHox3, an essential gene for embryonic development in Nilaparvata lugens. Arch. Insect Biochem. Physiol..

[13] Yu J.-L., An Z.-F., Liu X.-D. (2014). Wingless gene cloning and its role in manipulating the wing dimorphism in the white-backed planthopper, Sogatella furcifera. BMC Mol. Boil..

[14] Amiri A., Bandani A.R., Alizadeh H. (2015). Molecular identification of cysteine and trypsin protease, effect of different hosts on protease expression, and RNAi mediated silencing of cysteine protease gene in the sunn pest. Arch. Insect Biochem. Physiol..

[15] Coleman A.D., Wouters R.H.M., Mugford S.T., Hogenhout S.A. (2014). Persistence and transgenerational effect of plant-mediated RNAi in aphids. J. Exp. Bot..

[16] Shang F., Niu J.-Z., Ding B.-Y., Zhang Q., Ye C., Zhang W., Smagghe G., Wang J.-J. (2017). Vitellogenin and its receptor play essential roles in the development and reproduction of the brown citrus aphid, *Aphis* (*Toxoptera*) *citricidus*. Insect Mol. Boil..

[17] Yu N., Christiaens O., Liu J., Niu J., Cappelle K., Caccia S., Huvenne H., Smagghe G. (2012). Delivery of dsRNA for RNAi in insects: An overview and future directions. Insect Sci..

[18] Will T., Vilcinskas A. (2015). The structural sheath protein of aphids is required for phloem feeding. Insect Biochem. Mol. Boil..

[19] Ghanim M., Kontsedalov S., Czosnek H. (2007). Tissue-specific gene silencing by RNA interference in the whitefly Bemisia tabaci (Gennadius). Insect Biochem. Mol. Boil..

[20] Rosa C., Kamita S.G., Falk B.W. (2012). RNA interference is induced in the glassy winged sharpshooter Homalodisca vitripennis by actin dsRNA. Pest Manag. Sci..

[21] Joga M.R., Zotti M.J., Smagghe G., Christiaens O. (2016). RNAi Efficiency, Systemic Properties, and Novel Delivery Methods for Pest Insect Control: What We Know So Far. Front. Physiol..

[22] Katoch R., Sethi A., Thakur N., Murdock L.L. (2013). RNAi for Insect Control: Current Perspective and Future Challenges. Appl. Biochem. Biotechnol..

[23] Zhang H., Li H.-C., Miao X.-X. (2012). Feasibility, limitation and possible solutions of RNAi-based technology for insect pest control. Insect Sci..

[24] Chen J., Zhang D., Yao Q., Zhang J., Dong X., Tian H., Zhang W. (2010). Feeding-based RNA interference of atrehalose phosphate synthasegene in the brown planthopper, *Nilaparvata lugens*. Insect Mol. Boil..

[25] Price D.R., Gatehouse J.A. (2008). RNAi-mediated crop protection against insects. Trends Biotechnol..

[26] Guo L., Xie W., Liu Y., Yang Z., Yang X., Xia J., Wang S., Wu Q., Zhang Y. (2018). Identification and characterization of doublesex in *Bemisia tabaci*. Insect Mol. Boil..

[27] Fan J., Zhang Y., Francis F., Cheng D., Sun J., Chen J.-L. (2015). Orco mediates olfactory behaviors and winged morph differentiation induced by alarm pheromone in the grain aphid, *Sitobion avenae*. Insect Biochem. Mol. Boil..

[28] Vyas M., Raza A., Ali M.Y., Ashraf M.A., Mansoor S., Shahid A.A., Brown J.K. (2017). Knock down of Whitefly Gut Gene Expression and Mortality by Orally Delivered Gut Gene-Specific dsRNAs. PLoS ONE.

[29] Upadhyay S.K., Chandrashekar K., Thakur N., Verma P.C., Borgio J.F., Singh P.K., Tuli R. (2011). RNA interference for the control of whiteflies (*Bemisia tabaci*) by oral route. J. Biosci..

[30] Tariq K., Ali A., Davies T.G.E., Naz E., Naz L., Sohail S., Hou M., Ullah F. (2019). RNA interference-mediated knockdown of voltage-gated sodium channel (*MpNav*) gene causes mortality in peach-potato aphid, *Myzus persicae*. Sci. Rep..

[31] Araujo R., Santos A., Pinto F., Gontijo N.F., Lehane M., Pereira M.H. (2006). RNA interference of the salivary gland nitrophorin 2 in the triatomine bug *Rhodnius prolixus* (Hemiptera: Reduviidae) by dsRNA ingestion or injection. Insect Biochem. Mol. Boil..

[32] Christiaens O., Swevers L., Smagghe G. (2014). DsRNA degradation in the pea aphid (*Acyrthosiphon pisum*) associated with lack of response in RNAi feeding and injection assay. Peptides.

[33] Cooper A.M.W., Silver K., Zhang J., Park Y., Zhu K.Y. (2018). Molecular mechanisms influencing efficiency of RNA interference in insects. Pest Manag. Sci..

[34] Castellanos N.L., Smagghe G., Sharma R., Oliveira E.E., Christiaens O. (2018). Liposome encapsulation and EDTA formulation of dsRNA targeting essential genes increase oral RNAi-caused mortality in the Neotropical stink bug *Euschistus heros*. Pest Manag. Sci..

[35] Whyard S., Singh A.D., Wong S. (2009). Ingested double-stranded RNAs can act as species-specific insecticides. Insect Biochem. Mol. Boil..

[36] Niu J., Yang W., Tian Y., Fan J., Ye C., Shang F., Ding B., Zhang J., An X., Yang L. (2019). Topical dsRNA delivery induces gene silencing and mortality in the pea aphid. Pest Manag. Sci..

[37] Killiny N., Hajeri S., Tiwari S., Gowda S., Stelinski L.L. (2014). Double-Stranded RNA Uptake through Topical Application, Mediates Silencing of Five *CYP4* Genes and Suppresses Insecticide Resistance in *Diaphorina citri*. PLoS ONE.

[38] Zheng Y., Hu Y., Yan S., Zhou H., Song D., Yin M., Shen J., Songm D. (2019). A polymer/detergent formulation improves dsRNA penetration through the body wall and RNAi-induced mortality in the soybean aphid *Aphis glycines*. Pest Manag. Sci..

[39] Thairu M.W., Skidmore I.H., Bansal R., Nováková E., Hansen T.E., Li-Byarlay H., Wickline S.A., Hansen A.K. (2017). Efficacy of RNA interference knockdown using aerosolized short interfering RNAs bound to nanoparticles in three diverse aphid species. Insect Mol. Boil..

[40] Hunter W.B., Glick E., Paldi N., Bextine B.R. (2012). Advances in RNA interference: dsRNA Treatment in Trees and Grapevines for Insect Pest Suppression. Southwest Èntomol..

[41] Ghosh S.K.B., Hunter W.B., Park A.L., Gundersen-Rindal D.E. (2017). Double strand RNA delivery system for plant-sap-feeding insects. PLoS ONE.

[42] Rodrigues T.B., Figueira A., Abdurakhmonov I.Y. (2016). Management of insect pest by RNAi—A new tool for crop protection. RNA Interference.

[43] Powell M., Pyati P., Cao M., Bell H., Gatehouse J.A., Fitches E.C. (2017). Insecticidal effects of dsRNA targeting the *Diap1* gene in dipteran pests. Sci. Rep..

[44] Riga M., Denecke S., Livadaras I., Geibel S., Nauen R., Vontas J. (2019). Development of efficient RNAi in *Nezara viridula* for use in insecticide target discovery. Arch. Insect Biochem. Physiol..

[45] Vélez A.M., Fishilevich E., Matz N., Storer N.P., Narva K., Siegfried B.D. (2016). Parameters for Successful Parental RNAi as An Insect Pest Management Tool in Western Corn Rootworm, *Diabrotica virgifera virgifera*. Genes.

[46] Pinzón N., Bertrand S., Subirana L., Busseau I., Escriva H., Seitz H. (2019). Functional lability of RNA-dependent RNA polymerases in animals. PLoS Genet..

[47] Zha W., Peng X., Chen R., Du B., Zhu L., He G. (2011). Knockdown of Midgut Genes by dsRNA-Transgenic Plant-Mediated RNA Interference in the Hemipteran Insect *Nilaparvata lugens*. PLoS ONE.

[48] Scott J.G., Michel K., Bartholomay L.C., Siegfried B.D., Hunter W.B., Smagghe G., Zhu K.Y., Douglas A.E. (2013). Towards the elements of successful insect RNAi. J. Insect Physiol..

[49] Jaubert-Possamai S., Le Trionnaire G., Bonhomme J., Christophides G.K., Rispe C., Tagu D. (2007). Gene knockdown by RNAi in the pea aphid *Acyrthosiphon pisum*. BMC Biotechnol..

[50] Allen M.L., Walker W.B. (2012). Saliva of *Lygus lineolaris* digests double stranded ribonucleic acids. J. Insect Physiol..

[51] Ghodke A.B., Good R.T., Golz J.F., Russell D.A., Edwards O., Robin C. (2019). Extracellular endonucleases in the midgut of *Myzus persicae* may limit the efficacy of orally delivered RNAi. Sci. Rep..

[52] Luo Y., Chen Q., Luan J., Chung S.H., Van Eck J., Turgeon R., Douglas A.E. (2017). Towards an understanding of the molecular basis of effective RNAi against a global insect pest, the whitefly *Bemisia tabaci*. Insect Biochem. Mol. Boil..

[53] Dowling D., Pauli T., Donath A., Meusemann K., Podsiadlowski L., Petersen M., Peters R.S., Mayer C., Liu S., Zhou X. (2016). Phylogenetic Origin and Diversification of RNAi Pathway Genes in Insects. Genome Boil. Evol..

[54] Tomoyasu Y., Miller S.C., Tomita S., Schoppmeier M., Grossmann D., Bucher G. (2008). Exploring systemic RNA interference in insects: A genome-wide survey for RNAi genes in Tribolium. Genome Boil..

[55] Ye C., An X., Jiang Y.-D., Ding B.-Y., Shang F., Christiaens O., Taning C.N.T., Smagghe G., Niu J., Wang J.-J. (2019). Induction of RNAi Core Machinery’s Gene Expression by Exogenous dsRNA and the Effects of Pre-exposure to dsRNA on the Gene Silencing Efficiency in the Pea Aphid (*Acyrthosiphon pisum*). Front. Physiol..

[56] Bansal R., Michel A.P. (2013). Core RNAi Machinery and Sid1, a Component for Systemic RNAi, in the Hemipteran Insect, Aphis glycines. Int. J. Mol. Sci..

[57] Upadhyay S.K., Dixit S., Sharma S., Singh H., Kumar J., Verma P.C., Chandrashekar K. (2013). siRNA Machinery in Whitefly (*Bemisia tabaci*). PLoS ONE.

[58] Nicholson S.J., Nickerson M.L., Dean M., Song Y., Hoyt P., Rhee H., Kim C., Puterka G.J. (2015). The genome of *Diuraphis noxia*, a global aphid pest of small grains. BMC Genomics.

[59] Taning C.N.T., De Andrade E.C., Hunter W.B., Christiaens O., Smagghe G. (2016). Asian *Citrus Psyllid* RNAi Pathway—RNAi evidence. Sci. Rep..

[60] Sparks M.E., Shelby K.S., Kuhar D., Gundersen-Rindal D.E. (2014). Transcriptome of the Invasive Brown Marmorated Stink Bug, *Halyomorpha halys* (Stål) (Heteroptera: Pentatomidae). PLoS ONE.

[61] Davis-Vogel C., Van Allen B., Van Hemert J.L., Sethi A., Nelson M.E., Sashital D.G. (2018). Identification and comparison of key RNA interference machinery from western corn rootworm, fall armyworm, and southern green stink bug. PLoS ONE.

[62] Xu J., Xu X., Zhan S., Huang Y.P. (2019). Genome editing in insects: Current status and challenges. Natl. Sci. Rev..

[63] Swevers L., Smagghe G. (2012). Use of RNAi for Control of Insect Crop Pests.

[64] Dietzl G., Chen D., Schnorrer F., Su K.-C., Barinova Y., Fellner M., Gasser B., Kinsey K., Oppel S., Scheiblauer S. (2007). A genome-wide transgenic RNAi library for conditional gene inactivation in Drosophila. Nature.

[65] Lozano J., Gómez-Orte E., Lee H.-J., Bellés X., Lozano-Fernandez J. (2012). Super-induction of Dicer-2 expression by alien double-stranded RNAs: An evolutionary ancient response to viral infection?. Dev. Genes Evol..

[66] Sun Y., Sparks C., Jones H., Riley M., Francis F., Du W., Xia L. (2019). Silencing an essential gene involved in infestation and digestion in grain aphid through plant-mediated RNA interference generates aphid-resistant wheat plants. Plant Biotechnol. J..

[67] Matsumoto Y., Hattori M. (2016). Gene silencing by parental RNA interference in the green rice leafhopper, *Nephotettix cincticeps* (hemiptera: Cicadellidae). Arch. Insect Biochem. Physiol..

[68] Fishilevich E., Vélez A.M., Khajuria C., Frey M.L., Hamm R.L., Wang H., Schulenberg G.A., Bowling A.J., Pence H.E., Gandra P. (2016). Use of chromatin remodeling ATPases as RNAi targets for parental control of western corn rootworm (*Diabrotica virgifera virgifera*) and Neotropical brown stink bug (*Euschistus heros*). Insect Biochem. Mol. Boil..

[69] Brito T., Julio A., Berni M., Poncio L.D.C., Bernardes E.S., Araújo H.M.M., Sammeth M., Pane A. (2018). Transcriptomic and functional analyses of the piRNA pathway in the Chagas disease vector *Rhodnius prolixus*. PLoS Negl. Trop. Dis..

[70] Will T., Schmidtberg H., Skaljac M., Vilcinskas A. (2016). Heat shock protein 83 plays pleiotropic roles in embryogenesis, longevity, and fecundity of the pea aphid *Acyrthosiphon pisum*. Dev. Genes Evol..

[71] Simonet P., Gaget K., Parisot N., Duport G., Rey M., Febvay G., Charles H., Callaerts P., Colella S., Calevro F. (2016). Disruption of phenylalanine hydroxylase reduces adult lifespan and fecundity, and impairs embryonic development in parthenogenetic pea aphids. Sci. Rep..

[72] Bewick A.J., Sanchez Z., McKinney E.C., Moore A.J., Moore P.J., Schmitz R.J. (2019). Dnmt1 is essential for egg production and embryo viability in the large milkweed bug, *Oncopeltus fasciatus*. Epigenet. Chromatin.

[73] Panfilio K.A. (2009). Late extraembryonic morphogenesis and its zenRNAi-induced failure in the milkweed bug *Oncopeltus fasciatus*. Dev. Boil..

[74] Walter-Nuno A.B., Oliveira M.P., Oliveira M.F., Gonçalves R.L., Ramos I.B., Koerich L.B., Oliveira P.L., Paiva-Silva G.O. (2013). Silencing of Maternal Heme-binding Protein Causes Embryonic Mitochondrial Dysfunction and Impairs Embryogenesis in the Blood Sucking Insect *Rhodnius prolixus*. J. Boil. Chem..

[75] Matsuura Y., Kikuchi Y., Miura T., Fukatsu T. (2015). Ultrabithorax is essential for bacteriocyte development. Proc. Natl. Acad. Sci. USA.

[76] Chesebro J., Hrycaj S., Mahfooz N., Popadic A. (2009). Diverging functions of Scr between embryonic and post-embryonic development in a hemimetabolous insect, *Oncopeltus fasciatus*. Dev. Biol..

[77] Yan T., Chen H., Sun Y., Yu X.-D., Xia L. (2016). RNA Interference of the Ecdysone Receptor Genes EcR and USP in Grain Aphid (*Sitobion avenae*) Affects Its Survival and Fecundity upon Feeding on Wheat Plants. Int. J. Mol. Sci..

[78] Lu Y., Chen M., Reding K., Pick L. (2017). Establishment of molecular genetic approaches to study gene expression and function in an invasive hemipteran, *Halyomorpha halys*. Evodevo.

[79] Li X., Liu F., Wu C., Zhao J., Cai W., Hua H. (2019). Decapentaplegic function in wing vein development and wing morph transformation in brown planthopper, *Nilaparvata lugens*. Dev. Boil..

[80] Lin X., Yao Y., Jin M., Li Q. (2014). Characterization of the Distal-less gene homologue, NlDll, in the brown planthopper, *Nilaparvata lugens* (Stål). Gene.

[81] Wu W.-J., Wang Y., Huang H.-J., Bao Y.-Y., Zhang C. (2012). Ecdysone receptor controls wing morphogenesis and melanization during rice planthopper metamorphosis. J. Insect Physiol..

[82] El-Shesheny I., Hajeri S., El-Hawary I., Gowda S., Killiny N. (2013). Silencing Abnormal Wing Disc Gene of the Asian Citrus Psyllid, *Diaphorina citri* Disrupts Adult Wing Development and Increases Nymph Mortality. PLoS ONE.

[83] Xu N., Chen H., Xue W., Yuan X., Xia P., Xu H.-J. (2019). The MTase15 regulates reproduction in the wing-dimorphic planthopper, *Nilaparvata lugens* (Hemiptera: Delphacidae). Insect Mol. Boil..

[84] Ge L., Huang B., Jiang Y.-P., Gu H.-T., Xia T., Yang G.-Q., Liu F., Wu J.-C. (2017). Carboxylesterase Precursor (EST-1) Mediated the Fungicide Jinggangmycin-Suppressed Reproduction of *Sogatella furcifera* (Hemiptera: Delphacidae). J. Econ. Èntomol..

[85] Blariza M.J., Grosso C.G., García B.A. (2017). Silencing of Two Vitellogenin Genes Inhibits Oviposition in the Chagas Disease Vector *Triatoma infestans* (Hemiptera: Reduviidae). Am. J. Trop. Med. Hyg..

[86] Basnet S., Kamble S.T. (2017). Knockdown of the Chromatin Remodeling Gene Brahma by RNA Interference Reduces Reproductive Fitness and Lifespan in Common Bed Bug (Hemiptera: Cimicidae). J. Med Èntomol..

[87] Smykal V., Bajgar A., Provazník J., Fexova S., Buricova M., Takaki K., Hodkova M., Jindra M., Dolezel D. (2014). Juvenile hormone signaling during reproduction and development of the linden bug, *Pyrrhocoris apterus*. Insect Biochem. Mol. Boil..

[88] Jiang Y.-D., Yuan X., Bai Y.-L., Wang G.-Y., Zhou W.-W., Zhu Z.-R. (2018). Knockdown of timeless Disrupts the Circadian Behavioral Rhythms in *Laodelphax striatellus* (Hemiptera: Delphacidae). Environ. Èntomol..

[89] Lan H., Hong X., Huang R., Lin X., Li Q., Li K., Zhou T. (2017). RNA interference-mediated knockdown and virus-induced suppression of Troponin C gene adversely affect the behavior or fitness of the green rice leafhopper, *Nephotettix cincticeps*. Arch. Insect Biochem. Physiol..

[90] Zhang X., Pengsakul T., Tukayo M., Yu L., Fang W., Luo D. (2017). Host-location behavior of the tea green leafhopper *Empoasca vitis Göthe* (Hemiptera: Cicadellidae): Olfactory and visual effects on their orientation. Bull. Èntomol. Res..

[91] Li X., Qu M.-J., Zhang Y., Li J.-W., Liu T. (2018). Expression of Neuropeptide F Gene and Its Regulation of Feeding Behavior in the Pea Aphid, *Acyrthosiphon pisum*. Front. Physiol..

[92] Paim R.M.M., Araujo R., Lehane M.J., Gontijo N.F., Pereira M.H. (2013). Long-term effects and parental RNAi in the blood feeder *Rhodnius prolixus* (Hemiptera; Reduviidae). Insect Biochem. Mol. Boil..

[93] Ikeno T., Ishikawa K., Numata H., Goto S.G. (2013). Circadian clock gene Clockis involved in the photoperiodic response of the bean bugRiptortus pedestris. Physiol. Èntomol..

[94] Zhang Y., Li H., Du J., Zhang J., Shen J., Cai W. (2019). Three Melanin Pathway Genes, *TH*, *yellow*, and *aaNAT*, Regulate Pigmentation in the Twin-Spotted Assassin Bug, *Platymeris biguttatus* (Linnaeus). Int. J. Mol. Sci..

[95] Sun Z.-J., Li Z.-X. (2018). The terpenoid backbone biosynthesis pathway directly affects the biosynthesis of alarm pheromone in the aphid. Insect Mol. Boil..

[96] Fawcett M.M., Parks M.C., Tibbetts A.E., Swart J.S., Richards E., Vanegas J.C., Cenzer M.L., Crowley L., Simmons W.R., Hou W.S. (2018). Manipulation of insulin signaling phenocopies evolution of a host-associated polyphenism. Nat. Commun..

[97] Yin Z.-J., Dong X.-L., Kang K., Chen H., Dai X.-Y., Wu G.-A., Zheng L., Yu Y., Zhai Y.-F. (2018). FoxO Transcription Factor Regulate Hormone Mediated Signaling on Nymphal Diapause. Front. Physiol..

[98] Zhang Y., Zhu X., Gu S.-H., Zhou Y., Wang S., Guo Y.-Y. (2016). Silencing of odorant binding protein gene AlinOBP4 by RNAi induces declining electrophysiological responses of *Adelphocoris lineolatus* to six semiochemicals. Insect Sci..

[99] Walker W.B., Allen M.L. (2010). Expression and RNA Interference of Salivary Polygalacturonase Genes in the Tarnished Plant Bug, *Lygus lineolaris*. J. Insect Sci..

[100] Brent C.S., Hull J.J. (2018). RNA interference-mediated knockdown of eye coloration genes in the western tarnished plant bug (*Lygus hesperus Knight*). Arch. Insect Biochem. Physiol..

[101] Luo J., Li Z., Ma C., Zhang Z., Hull J.J., Lei C., Jin S., Chen L. (2017). Knockdown of a metathoracic scent gland desaturase enhances the production of (E)-4-oxo-2-hexenal and suppresses female sexual attractiveness in the plant bug *Adelphocoris suturalis*. Insect Mol. Boil..

[102] Nishide Y., Kageyama D., Yokoi K., Jouraku A., Tanaka H., Futahashi R., Fukatsu T. (2019). Functional crosstalk across IMD and Toll pathways: Insight into the evolution of incomplete immune cascades. Proc. R. Soc. B.

[103] Wang Z.-Z., Bing X.-L., Liu S.-S., Chen X.-X. (2016). RNA interference of an antimicrobial peptide, Btdef, reduces Tomato yellow leaf curl China virus accumulation in the whitefly *Bemisia tabaci*. Pest Manag. Sci..

[104] Eliautout R., Dubrana M.-P., Vincent-Monégat C., Vallier A., Braquart-Varnier C., Poirié M., Saillard C., Heddi A., Arricau-Bouvery N. (2016). Immune response and survival of *Circulifer haematoceps* to *Spiroplasma citri* infection requires expression of the gene hexamerin. Dev. Comp. Immunol..

[105] Chen Y., Redinbaugh M.G., Michel A.P. (2015). Molecular interactions and immune responses between Maize fine streak virus and the leafhopper vector *Graminella nigrifrons* through differential expression and RNA interference. Insect Mol. Boil..

[106] Chen Q., Zheng L., Mao Q., Liu J., Wang H., Jia D., Chen H., Wu W., Wei T. (2019). Fibrillar structures induced by a plant reovirus target mitochondria to activate typical apoptotic response and promote viral infection in insect vectors. PLoS Pathog..

[107] Mao K., Zhang X., Ali E., Liao X., Jin R., Ren Z., Wan H., Li J. (2019). Characterization of nitenpyram resistance in *Nilaparvata lugens* (Stål). Pestic. Biochem. Physiol..

[108] Tian F., Li C., Wang Z., Liu J., Zeng X. (2018). Identification of detoxification genes in imidacloprid-resistant *Asian citrus psyllid* (Hemiptera: Lividae) and their expression patterns under stress of eight insecticides. Pest Manag. Sci..

[109] Chen X., Xia J., Shang Q., Song D., Gao X. (2019). UDP-glucosyltransferases potentially contribute to imidacloprid resistance in *Aphis gossypii* glover based on transcriptomic and proteomic analyses. Pestic. Biochem. Physiol..

[110] Gong Y.-H., Yu X.-R., Shang Q.-L., Shi X.-Y., Gao X.-W. (2014). Oral Delivery Mediated RNA Interference of a Carboxylesterase Gene Results in Reduced Resistance to Organophosphorus Insecticides in the Cotton Aphid, *Aphis gossypii Glover*. PLoS ONE.

[111] Xiao D., Lu Y.-H., Shang Q., Song D.-L., Gao X. (2014). Gene silencing of two acetylcholinesterases reveals their cholinergic and non-cholinergic functions in *Rhopalosiphum padi* and *Sitobion avenae*. Pest Manag. Sci..

[112] Thakur N., Mundey J.K., Upadhyay S.K., Abdurakhmonov I.Y. (2016). RNAi—Implications in Entomological Research and Pest Control. RNA Interference.

[113] Abdellatef E., Will T., Koch A.M., Imani J., Vilcinskas A., Kogel K.-H. (2015). Silencing the expression of the salivary sheath protein causes transgenerational feeding suppression in the aphid Sitobion avenae. Plant Biotechnol. J..

[114] Pick L. (2015). Hox genes, evo-devo, and the case of the ftz gene. Chromosoma.

[115] Angelini D.R., Liu P.Z., Hughes C.L., Kaufman T.C. (2005). Hox gene function and interaction in the milkweed bug *Oncopeltus fasciatus* (Hemiptera). Dev. Boil..

[116] Liu P.Z., Kaufman T.C. (2004). hunchback is required for suppression of abdominal identity, and for proper germband growth and segmentation in the intermediate germband insect *Oncopeltus fasciatus*. Development.

[117] Terenius O., Papanicolaou A., Garbutt J.S., Eleftherianos I., Huvenne H., Kanginakudru S., Albrechtsen M., An C., Aymeric J.-L., Barthel A. (2011). RNA interference in Lepidoptera: An overview of successful and unsuccessful studies and implications for experimental design. J. Insect Physiol..

[118] Sapountzis P., Duport G., Balmand S., Gaget K., Jaubert-Possamai S., Febvay G., Charles H., Rahbé Y., Colella S., Calevro F. (2014). New insight into the RNA interference response against cathepsin-L gene in the pea aphid, *Acyrthosiphon pisum*: Molting or gut phenotypes specifically induced by injection or feeding treatments. Insect Biochem. Mol. Boil..

[119] Ye C., Jiang Y.-D., An X., Yang L., Shang F., Niu J., Wang J.-J. (2019). Effects of RNAi-based silencing of chitin synthase gene on moulting and fecundity in pea aphids (*Acyrthosiphon pisum*). Sci. Rep..

[120] Perrimon N., Ni J.-Q., Perkins L. (2010). In vivo RNAi: Today and Tomorrow. Cold Spring Harb. Perspect. Boil..

[121] Yang Y., Wan P.-J., Hu X.-X., Li G.-Q. (2014). RNAi mediated knockdown of the ryanodine receptor gene decreases chlorantraniliprole susceptibility in *Sogatella furcifera*. Pestic. Biochem. Physiol..

[122] Scott J.G. (1990). Investigating Mechanisms of Insecticide Resistance: Methods, Strategies, and Pitfalls. Pesticide Resistance in Arthropods.

[123] Ding B.-Y., Shang F., Zhang Q., Xiong Y., Yang Q., Niu J., Smagghe G., Wang J.-J. (2017). Silencing of Two Insulin Receptor Genes Disrupts Nymph-Adult Transition of *Alate Brown Citrus Aphid*. Int. J. Mol. Sci..

[124] Moriyama M., Hosokawa T., Tanahashi M., Nikoh N., Fukatsu T. (2016). Suppression of Bedbug’s Reproduction by RNA Interference of Vitellogenin. PLoS ONE.

[125] Chen J., Zhang D.W., Jin X., Xu X.L., Zeng B.P. (2018). Characterization of the akirin gene and its role in the NF-kappaB signaling pathway of *Sogatella furcifera*. Front. Physiol..

[126] Peng T., Pan Y., Yang C., Gao X., Xi J., Wu Y., Huang X., Zhu E., Xin X., Zhan C. (2016). Over-expression of *CYP6A2* is associated with spirotetramat resistance and cross-resistance in the resistant strain of *Aphis gossypii Glover*. Pestic. Biochem. Physiol..

